# Anti-ulcerogenic activity of the root bark extract of the African laburnum *“Cassia sieberiana”* and its effect on the anti-oxidant defence system in rats

**DOI:** 10.1186/1472-6882-12-247

**Published:** 2012-12-10

**Authors:** Edmund T Nartey, Mark Ofosuhene, Caleb M Agbale

**Affiliations:** 1 Centre for Tropical Clinical Pharmacology and Therapeutics, University of Ghana Medical School, P.O. Box GP 4236, Accra, Ghana; 2 Department of Clinical Pathology, Noguchi Memorial Institute for Medical Research, University of Ghana, P. O. Box LG 581, Legon, Ghana; 3 Department of Biochemistry, School of Biological Sciences, University of Cape Coast, Cape Coast, Ghana

**Keywords:** Gastric ulcer, *Cassia sieberiana*, Superoxide dismutase, Catalase, Glutathione peroxidase, Lipid hydroperoxide, Myeloperoxidase

## Abstract

**Background:**

Despite the widespread use of roots of *Cassia sieberiana* in managing several health conditions including gastric ulcer disease, there is little scientific data to support the rational phytotherapeutics as an anti-ulcer agent. This paper reports an evaluation of the *in vivo* anti-oxidant properties of an aqueous root bark extract of *C. sieberiana* in experimental gastric ulcer rats in a bid to elucidate its mechanism of action.

**Methods:**

Fisher 344 (F_344_) rats received pretreatment of *C. sieberiana* root bark extract (500, 750, and 1000 mg/kg body wt.) for 7 days after which there was induction of gastric injury with absolute ethanol. The mean ulcer index (MUI) was calculated and serum total anti-oxidant level determined. Gastric mucosal tissues were prepared and the activity level of the enzymes superoxide dismutase (SOD), catalase (CAT), glutathione peroxidase (GPx) and myeloperoxidase (MPO) were measured together with the level of lipid hydroperoxides (LPO). Statistical difference between treatment groups was analysed using one-way analysis of variance (ANOVA) followed by Dunnett’s *post hoc t* test. Statistical significance was calculated at P< 0.05.

**Results:**

The administration of ethanol triggered severe acute gastric ulcer and pretreatment with *C. sieberiana* root bark extract significantly and dose dependently protected against this effect. The root bark extract also dose dependently and significantly inhibited the ethanol induced decrease in activity levels of the enzymes SOD, CAT and GPx. The extract also inhibited the ethanol-induced decrease in level of serum total anti-oxidant capacity. The increase in ethanol-induced LPO level and MPO activity were also significantly and dose-dependently inhibited by the root bark extract.

**Conclusions:**

The gastro-cytoprotective effect, inhibition of decrease in activity of gastric anti-oxidant enzymes and MPO as well as the inhibition of gastric LPO level suggests that one of the anti-ulcer mechanisms of *C. sieberiana* is the anti-oxidant property.

## Background

Gastric ulcer disease (GUD) is a common problem of the gastro-intestinal tract with increasing incidence and prevalence attributed to various factors including the widespread use of non-steroidal anti-inflammatory drugs (NSAIDs). GUD is reported to affect about 14.5 million people worldwide with a mortality of about 4.08 million [1]. Although the etiology of gastric ulcers is still debated, it is accepted that gastric ulcers are triggered by an imbalance between factors that damage and those that protect the stomach. Mucosal damage, an initial step in gastric ulcer development, has been known to be due to hypersecretion of HCl through H^+^, K^+^-ATPase action [2], harbouring of *H. pylori* on the damaged mucin layer [3], the blockade of the cyclooxygenase enzyme system by NSAIDs [4], and oxidative stress by reactive oxygen species.

Reactive oxygen species (ROS) and free radicals play an important role in the pathogenesis of several human diseases including GUD [5,6]. Various studies have also shown that the endogenous anti-oxidant defense enzymes play a principal role in eliminating ROS and free radicals generated from the action of factors that damage the stomach. An approach to manage GUD, therefore, is through the scavenging of ROS and the stimulation of the endogenous anti-oxidant enzymes in the stomach in addition to the other approaches like the inhibition of gastric H^+^, K^+^-ATPase and the elimination of *H. pylori* using antibiotics.

*Cassia sieberiana* (*Cassia kotschyana* Oliv.; Fam. Caesalpinaceae), is a savannah tree that grows to about 15 m tall and is commonly fairly cultivated because of its attractive blossom and curious fruits (commonly referred to as the African laburnum). *C. sieberiana* has a very wide range of phytotherapeutic application in Ghana including the use of its roots in the management of hernia, leprosy, indigestion and gastric ulcer [7]. Studies by Nartey et al. [8] reported that the aqueous root bark extract of *C. sieberiana* possesses gastro-cytoprotective, anti-secretory and ulcer healing properties with the suggestion that its anti-ulcerogenic activity may be due to inhibition of the H^+^, K^+^-ATPase proton pump and increase in activity of gastric anti-oxidative enzymes. In another study by Nartey et al. [9], the aqueous root bark extract was shown to possess *in vitro* free radical scavenging and anti-oxidant properties and could stimulate the production of gastric mucosal prostaglandins E_2_ and I_2_. The aim of this study was therefore to assess the gastro-cytoprotective effect of root bark extract of *C. sieberiana* in comparison with ranitidine in a model of ethanol-induced gastric ulcer in rats and to elicit the underlying *in vivo* anti-oxidative mechanisms. For this purpose, the role of the root bark extract in oxidative stress was studied by measuring changes in activity levels of superoxide dismutase (SOD), catalase (CAT) and glutathione peroxidase (GPx). In addition, the total anti-oxidant capacity of blood serum, the level of lipid peroxidation (LPO) and the activity level of myeloperoxidase (MPO) as a marker of neutrophil infiltration of the stomach homogenates were also measured.

## Methods

### Preparation of root bark extract

Fresh roots of *C. sieberiana* (*Cassia kotschyana Oliv*.) were obtained from the grounds of the University of Ghana, authenticated at the Ghana herbarium and prepared as previously described by Nartey et al. [8]. Briefly, chopped fresh root barks of *C. sieberiana* (750 g) were grounded and mixed with 1L of water and left to stand for 24 hours. The resulting concoction was evaporated under reduced pressure with the rotary evaporator at 30°C and the concentrate freeze-dried to yield solid material which was stored at 4°C and used within 4 weeks of production.

### Animals

Fisher 344 (F_344_) rats weighing 230.3 ± 14.5 g (mean ± S.D) of both sexes were used for the studies. They were raised on standard laboratory diet (GAFCO, Tema, Ghana). The animal experimentation described in this study was approved by the Scientific and Technical Committee of the Noguchi Memorial Institute for Medical Research (NMIMR) of the University of Ghana and was conducted in accordance with internationally accepted principles for laboratory animal use and care.

### Chemicals and reagents

The enzyme immunoassay kits for total anti-oxidants (Item # 709001), lipid hydroperoxide-LPO (Item # 705002), superoxide dismutase-SOD (Item # 706002), catalase-CAT (Item # 707002) and glutathione peroxidase-GPx (Item # 703102), were obtained from Cayman Chemical Company (Ann Arbor, U.S.A). Protein assay kit (Item # 704002) was also obtained from Cayman Chemical Company (Ann Arbor, U.S.A). All other chemicals and reagents were obtained from Sigma-Aldrich Chemical Company, St. Louis, MO, U.S.A.

### Administration of plant extract and induction of gastric ulcers

Different concentrations of *C. sieberiana* root bark extract (dissolved in water as vehicle) were administered by oral gavage to the different animal groups (six animals per group) at 12 h intervals in a total volume of 2 mL for 7 days. The concentration of the plant extract was determined based on the earlier report of Nartey et al. [8]. One hour after the last administration of the root bark extract and after overnight fasting, animals received absolute ethanol (1 mL/200 g body weight) by the same route [10]. One hour after the administration of ethanol, the animals were euthanised and blood collected by cardiac puncture. Serum was separated and used for the determination of total anti-oxidant capacity. The stomachs were then removed, cut open along the greater curvature and flushed gently with lukewarm water. Thereafter, the gastric mucosa was observed for lesions with the aid of a magnifying glass and the severity of the mucosal lesions scored according to the method of Marhueda et al. [11]. The vehicle (water) and ranitidine (50 mg/kg body weight) were administered similarly as the *C. sieberiana* root bark extract. The ulcer score for each animal was determined and the mean ulcer index (MUI) and percentage inhibition for each group calculated as follows:

(1) $$\;\mathrm{MUI}=\frac{\mathrm{Total}\;\mathrm{ulcer}\;\mathrm{score}}{\mathrm{Number}\;\mathrm{of}\;\mathrm{rats}\;\mathrm{ulcerated}}$$

(2) $$\%\;\mathrm{Inhibition}=\frac{\mathrm{MUI}\;\mathrm{of}\;\mathrm{ethanol}\;\mathrm{treated}-\mathrm{MUI}\;\mathrm{of}\;\mathrm{extract}\;\mathrm{pretreated}}{\mathrm{UI}\;\mathrm{of}\;\mathrm{ethanol}\;\mathrm{treated}}\times 100$$

### Total antioxidant capacity

The capacity of total anti-oxidants in the serum was assayed as per instructions from Cayman Chemical Company (Ann Arbor, U.S.A). Briefly the blood was allowed to clot for 30 min and then centrifuged at 2,000 g for 15 min at 4°C. The resulting supernatant was assayed for total anti-oxidant capacity using Cayman’s anti-oxidant assay kit (Item # 709001).

### Determination of LPO levels and anti-oxidant enzymes activities

In other set of experiments, after euthanasia the stomachs were quickly removed, rinsed in phosphate buffered saline (PBS, pH 7.4) and weighed. The stomachs were then homogenized and centrifuged according to the standard procedures using ELISA kits from Cayman Chemical Company (Ann Arbor, U.S.A) and the supernatant assayed as per instructions for determination of LPO (Item # 705002) and the activity of the anti-oxidant enzymes SOD (Item # 706002), CAT (Item # 707002) and GPx (Item # 703102). Protein levels were estimated by protein assay kits (Item # 704002) from Cayman Chemical Company (Ann Arbor, U.S.A).

### Determination of myeloperoxidase (MPO) activity

In another set of experiment, immediately after euthanasia the stomachs were quickly removed, homogenized (1:10 wt/vol) in 0.5% hexadecyltrimethyl ammonium bromide (Sigma-Aldrich Chemical Company, U.S.A) in 50 mmol potassium phosphate buffer (pH 6.0) and assayed by the method of Bradley et al. [12] as described by Tariq et al. [13]. MPO activity in gastric tissues was expressed as μmol/min/mg tissue.

### Statistical analysis

Comparisons between means were performed using one-way analysis of variance (ANOVA) followed by Dunnett’s *post hoc t* test to determine statistical significance. P< 0.05 was considered significant.

## Results

### Anti-ulcer activity

**Figure 1:**
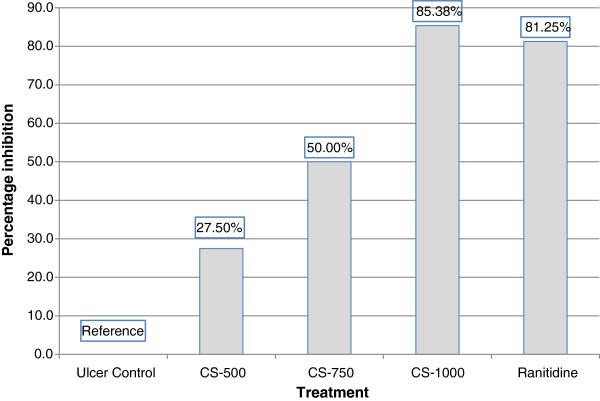
**Effect of *C. sieberiana *(CS) root bark extract on gastric ulcer inhibition in F_344_ rats.**

The result of the effect of ethanol on ulcer index is shown in Table 1. Treatment of rats with absolute ethanol produced extensive gastric ulcers in the glandular mucosa of stomach in all of the control animals. The mean ulcer index (MUI) was found to be 8.00 ± 0.37 in control animals (Table 1). Pretreatment of rats with *C. sieberiana* root bark extract in the doses of 500 mg/kg (MUI, 5.83 ± 0.31), 750 mg/kg (MUI, 4.00 ± 0.37), and 1000 mg/kg (MUI, 1.170 ± 0.17) for 7 days significantly and dose-dependently inhibited the formation of gastric lesions (p< 0.001). There were 27.50%, 50.00% and 85.38% protection in the CS-500, CS-750 and CS-1000 groups respectively (Figure 1). There was no significant difference (p> 0.05) in ulcer inhibition between the ranitidine pre-treated control group (81.25%) and the CS-1000 group (85.38%).

**Table 1:** **Effect of *C. sieberiana *(CS) root bark extract on ulcer index in ethanol-induced gastric ulcer rats**

**Treatment**	**Dose (mg/kg body wt.)**	**Mean Ulcer index [Score 0–10](Mean ± SEM)**
Baseline (intact)	-	-
Ulcer control	Vehicle	8.00 ± 0.37
CS-extract	500	5.80 ± 0.31^a^
“	750	4.00 ± 0.37^b, c^
“	1000	1.17 ± 0.17^b, d^
Ranitidine	50	1.50 ± 0.22^b^

^a^ p< 0.05 compared with ulcer control; ^b^ p< 0.001 compared with ulcer control; ^c^ p< 0.05 compared with CS-500; ^d^ p< 0.01 compared with CS-750; Values are Mean ± SEM of 6 animals. Statistical significance was determined by one-way analysis of variance (ANOVA) followed by Dunnett’s *post hoc t* test.

### Lipid peroxidation index

Table 2 shows that treatment of animals with absolute ethanol induced the generation of significant amounts (p< 0.001) of LPO from 2.85 ± 0.17 nmol/mg protein in the baseline (intact) to 8.34 ± 0.13 nmol/mg protein in the ulcer control. Administration of the plant extract significantly (p< 0.001) inhibited the generation of lipid hydroperoxides in a dose-dependent manner. The high dose of *C. sieberiana* (CS-1000) inhibited the generation of LPO from 8.34 ± 0.13 nmol/mg protein in the ulcer control to 3.13 ± 0.13 nmol/mg protein which was significantly higher (p< 0.001) than the effect of ranitidine (5.09 ± 0.06 nmol/mg protein).

**Table 2:** **Effect of *C. sieberiana *(CS) root bark extract on lipid peroxidation indices in ethanol-induced gastric ulcer rats**

**Treatment**	**Dose (mg/kg body wt.)**	**LPO (nmol/mg protein)**	**MPO (μmol/min/mg tissue)**
Baseline (intact)	-	2.85 ± 0.17	2.80 ± 0.20
Ulcer control	Vehicle	8.34 ± 0.13^a^	9.20 ± 0.26^a^
CS-extract	500	6.68 ± 0.11^b^	6.13 ± 0.27^b^
“	750	5.30 ± 0.15^b, c^	4.49 ± 0.12^b, f^
“	1000	3.13 ± 0.17^b, d^	3.27 ± 0.10^b, d^
Ranitidine	50	5.09 ± 0.06^b, e^	4.22 ± 0.08^b, e^

^a^ p< 0.001 compared with baseline; ^b^ p< 0.001 compared with ulcer control; ^c^ p< 0.001 compared with CS-500; ^d^ p< 0.001 compared with CS-750; ^e^ p< 0.001 compared with CS-1000; ^f^ p< 0.05 compared with CS-500; Values are Mean ± SEM of 6 animals. Statistical significance was determined by one-way analysis of variance (ANOVA) followed by Dunnett’s *post hoc t* test.

### MPO activity

Table 2 shows that the activity of gastric MPO was significantly increased (p< 0.001) from 2.80 ± 0.20 μmol/min/mg tissue in the baseline (intact) group to 9.20 ± 0.26 μmol/min/mg tissue when the rats were administered ethanol. Pre-treatment with different doses of the extract significantly (p< 0.001) attenuated ethanol-induced increase in gastric MPO activity in rats in a dose-dependent manner which was similar to the effect of ranitidine. However the effect of the high dose of *C. sieberiana* root bark extract (CS-1000) (3.27 ± 0.10 μmol/min/mg tissue) was significantly higher (p< 0.001) than that of ranitidine (4.22 ± 0.08 μmol/min/mg tissue), the reference anti-ulcer agent.

### Serum total anti-oxidant capacity

**Figure 2:**
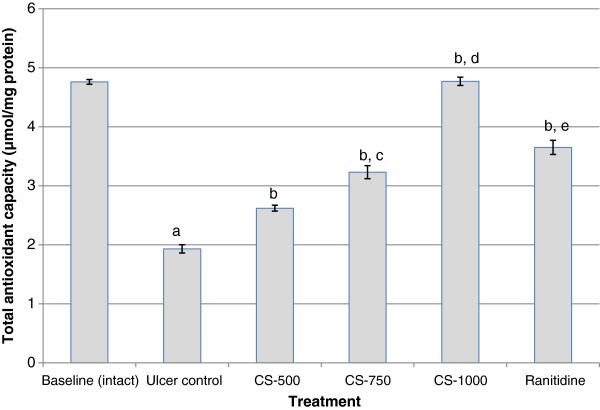
**Effect of *C. sieberiana *(CS) plant extract on serum total anti-oxidant capacity in F_344_ rats.**^a^ p<0.001 compared with baseline (intact); ^b^ p<0.001 compared with ulcer control; ^c^ p<0.05 compared with CS-500; ^d^ p<0.001 compared with CS-750; ^e^ p<0.01 compared with CS-1000; Values are Mean ± SEM of 6 animals. Statistical significance was determined by one-way analysis of variance (ANOVA) followed by Dunnett’s *post hoc t* test.

The results in Figure 2 show the effect of treatments on total anti-oxidant capacity in serum. Analysis of the results indicates that untreated animals induced with gastric ulcer showed a significant decrease (p< 0.001) in serum total anti-oxidants (1.93 ± 0.07 μmol/mg protein) when compared with the intact gastric mucosa (4.76 ± 0.04 μmol/mg protein). Animals pre-treated with ranitidine and extract at the three tested doses showed a significant dose-dependent increase in total serum anti-oxidants. This improvement was most pronounced (4.77 ± 0.07 μmol/mg protein) in the group that received the high dose of *C. sieberiana* root bark extract (CS-1000). Pre-treatment of animals with ranitidine, the standard anti-ulcer drug showed significantly lower total serum anti-oxidant capacity compared with CS-1000 (p< 0.01).

### SOD activity

**Figure 3:**
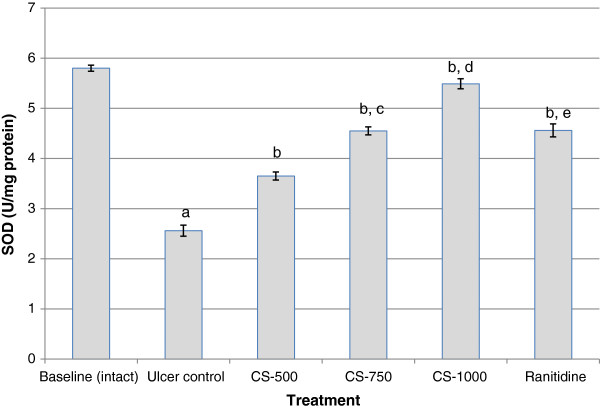
**Effect of *C. sieberiana *(CS) plant extract on mucosal superoxide dismutase activity in F_344_ rats.**^a^ p<0.001 compared with baseline (intact); ^b^ p<0.001 compared with ulcer control; ^c^ p<0.001 compared with CS-500; ^d^ p<0.001 compared with CS-750; ^e^ p<0.01 compared with CS-1000; Values are Mean ± SEM of 6 animals. Statistical significance was determined by one-way analysis of variance (ANOVA) followed by Dunnett’s *post hoc t* test.

The results in Figure 3 represent the effect of various treatments on SOD activity. Analysis of the figure shows that the activity of SOD was significantly lowered (p< 0.001) in the gastric mucosa of animals exposed to ethanol (Ulcer control) (2.56 ± 0.11 U/mg protein), when compared with the respective value in intact gastric mucosa (baseline) (5.80 ± 0.06 U/mg protein). Pre-treatment of the rats with *C. sieberiana* for 7 days showed a dose-dependent significant (p< 0.001) inhibition of the effect of ethanol in decreasing SOD. Pre-treatment with ranitidine also showed a significant (p< 0.001) inhibition of the effect of ethanol on gastric mucosal SOD but this was lower (p< 0.01) than the effect of CS-1000.

### CAT activity

**Figure 4:**
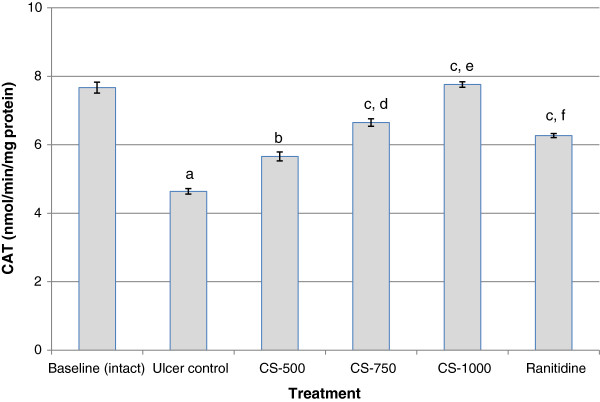
**Effect of *C. sieberiana *(CS) plant extract on mucosal catalase activity in F_344_ rats.**^a^ p<0.001 compared with baseline (intact); ^b^ p<0.01 compared with ulcer control; ^c^ p<0.001 compared with ulcer control; ^d^ p<0.01 compared with CS-500; ^e^ p<0.001 compared with CS-750; ^f^ p<0.001 compared with CS-1000; Values are Mean ± SEM of 6 animals. Statistical significance was determined by one-way analysis of variance (ANOVA) followed by Dunnett’s *post hoc t* test.

The results of the effect of different treatments on catalase activity are shown in Figure 4. The activity of catalase in gastric mucosa significantly varied after ethanol treatment, decreasing from 7.67 ± 0.16 nmol/min/mg protein in the baseline (intact) group to 4.64 ± 0.08 nmol/min/mg protein in the group exposed to ethanol (Ulcer control). Figure 4 also shows that pre-treatment of the animals with different doses of the root bark extract for 7 days significantly inhibited the effect of the ethanol on CAT in a dose-dependent manner. Rats administered 1000mg/kg bd. weight of the extract significantly increased (p< 0.001) CAT activity from 4.64 ± 0.08 nmol/min/mg protein in the ulcer control to 7.76 ± 0.08 nmol/min/mg protein in CS-1000. This increase in CAT activity was significantly higher (p< 0.001) than that recorded for ranitidine (6.27 ± 0.05 nmol/min/mg protein).

### GPx activity

**Figure 5:**
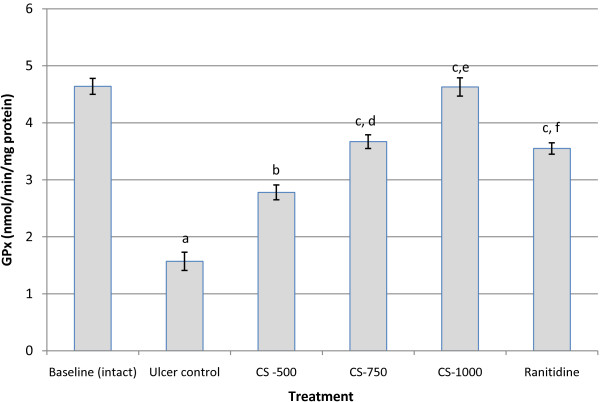
**Effect of *C. sieberiana *(CS) plant extract on mucosal glutathione peroxidase activity in F_344_ rats.**^a^ p<0.001 compared with baseline (intact); ^b^ p<0.01 compared with ulcer control; ^c^ p<0.001 compared with ulcer control; ^d^ p<0.01 compared with CS-500; ^e^ p<0.05 compared with CS-750; ^f^ p<0.01 compared with CS-1000; Values are Mean ± SEM of 6 animals. Statistical significance was determined by one-way analysis of variance (ANOVA) followed by Dunnett’s *post hoc t* test.

The effect of the extract and ranitidine, a reference compound on the activity of GPx are shown in Figure 5. The graph shows that the administration of ethanol significantly reduced (p< 0.001) the activity of GPx from 3.75 ± 0.16 nmol/min/mg protein in the baseline (intact) to 1.57 ± 0.16 nmol/min/mg protein in the ulcer control. All three doses of *C. sieberiana* significantly inhibited the effect of ethanol on GPx compared with the ulcer control. The CS-1000 succeeded in inducing a significant improvement (p< 0.001) in GPx activity compared with the ulcer control and this effect was similar but significantly higher (p< 0.01) to that of ranitidine.

## Discussion

Gastric ulcer disease is a multi-factorial disease and the significant role played by reactive oxygen species and free radicals [5] during its pathogenesis is well experimented in both human and experimental animals [14]. The administration of absolute ethanol in the induction of gastric ulcer in experimental rats is one of several methods used in the assessment of the gastro- cytoprotective action of anti-ulcer agents. The injurious effect of absolute ethanol in the stomach as a result of superficial aggressive necrosis due to enhanced lipid peroxidation also involves various mediators like lipoxygenase, cytokines [15], and excessive generation of oxygen-derived free radicals [16].

The present study was undertaken to investigate the *in-vivo* anti-oxidant effect of the root bark extract of *C. sieberiana* in a model of ethanol-induced gastric ulcer in rats. The results of the present study indicates that oral administration of *C. sieberiana* root bark extract had a significant and dose-dependent increase of the MUI inhibition (percent) against ethanol-induced gastric lesions (Figure 1). Compared with the untreated ulcer control animals, the inhibition of the gastric ulcers were 27.50%, 50.00% and 85.38% respectively for animals pretreated with 500 mg/kg, 750 mg/kg and 1000 mg/kg body weight of *C. sieberiana*. This data suggests that *C. sieberiana* root bark extract possesses strong gastro-cytoprotective properties against ethanol-induced gastric ulcers. This is in agreement with earlier reports that the root bark extract possesses anti-ulcerogenic properties in other induced gastric ulcer animal models [8] and is supported by the inhibition of decrease of the anti-oxidative enzymes SOD, CAT and GPx.

The endogenous anti-oxidant enzymes SOD, CAT and GPx in the gastric mucus are the key component of cellular defense system against reactive oxygen species. SOD catalyses the dismutation of O_2_^-^ to H_2_O_2_ which is then scavenged by CAT and GPx. The activity of GPx leads to the elimination of lipid hydroperoxides and hence plays an important role in cell protection [17]. Thus, inhibition of the activities of these enzymes in the gastric mucosa by ethanol may result in the accumulation of hydrogen peroxide with subsequent oxidation of lipids which will ultimately lead to loss of membrane integrity. Results from this study indicate that ethanol administration significantly decreased (p< 0.001) the activities of SOD, CAT and GPx and also serum total anti-oxidant capacity when compared with the un-ulcerated baseline animals. This observation was made 1 h after absolute ethanol administration. A decrease of SOD, CAT and GPx activities in gastric mucosa of rats exposed to ethanol leads to the accumulation of ROS and consequently to an increase in lipid peroxidation concentration and hence an increased mucosal damage as seen from the increase in MUI. These observations confirm the findings of several studies, which reported alterations in anti-oxidant enzyme activities in ethanol exposed animals [18-22]. The dose-dependent inhibition of ethanol-induced decrease in activity levels of gastric SOD, CAT and GPx when the animals where pretreated with *C. sieberiana* indicates that the root bark extract contains bioactive substances which can stimulate the activity of the endogenous gastric anti-oxidant enzyme system. The induced activity of the antioxidant defence system was supported by an increase in serum total antioxidant capacity, decrease in lipid hydroperoxides (LPO) and inhibition of myeloperoxidase (MPO) activity.

Induced SOD, CAT and GPx activities explains the decrease in LPO from 8.34 ± 0.13 nmol/mg protein in the ulcer control to 3.13 ± 0.13 nmol/mg protein in CS-1000 which is in correlation with the maintenance of baseline (intact) enzyme activity levels of SOD, CAT and GPx (p> 0.05). The gastro-cytoprotective effect of the root bark extract was also accompanied by inhibition of ethanol-induced increase in MPO from 9.20 ± 0.26 μmol/min/mg tissue in the ulcer control to 3.27 ± 0.10 μmol/min/mg tissue in CS-1000. The MPO assay is widely used as an index of neutrophil infiltration in various gastric ulcer models [23-25]. Neutrophils are the major inflammatory cell type infiltrating the injured mucosa following exposure to ethanol [26] and strategies to counteract the infiltration and/or activation of neutrophils have been shown to protect animals against gastric ulcers [27]. The effect of *C. sieberiana* in inhibiting an increase of MPO activity may therefore be related to its gastro-cytoprotective ability. This inhibition of neutrophil activation is in agreement with earlier studies that the root bark extract has anti-inflammatory properties [9,28].

The *in-vivo* anti-oxidant property of *C. sieberiana* root bark extract demonstrated in this study may be due to the presence of flavonol/flavonoid/flavone or related compounds with polyhydroxy and/or phenolic groups which we had earlier detected by phytochemical analysis [9]. Further studies we previously conducted [9] demonstrated that TLC spots revealed by fluorescence under UV light and their reaction with ferric chloride suggest that flavonol/flavonoid/flavone or related compounds with polyhydroxy and/or phenolic groups constituted major chemical substances in the root bark extract. Flavonoids or polyphenolic substances quench ROS, chelate metal ions and regenerate membrane-bound anti-oxidants and it has been demonstrated that substances with anti-oxidant properties, such as polyphenolic compounds, may protect against gastric-damaging effects caused by ethanol [29-35].

## Conclusion

In conclusion, the present results suggest that the gastro-cytoprotective effect of *C. sieberiana* root bark extract against mucosal injury induced by ethanol might be mediated at least partially by its ability to stimulate and thereby maintain baseline activity level of mucosal anti-oxidant enzymes such as SOD, CAT and GPx which constitute endogenous scavengers of ROS. This effect is supported by the elevation of serum total anti-oxidant level and the inhibition of increase in LPO in addition to the inhibition of MPO enzyme activity to maintain them at near basal level.

## Abbreviations

CS: *Cassia sieberiana*; CAT: Catalase; F_344_: Fisher 344; GPx: Glutathione peroxidase; LPO: Lipid hydroperoxide; MPO: Myeloperoxidase; MUI: Mean ulcer index; ROS: Reactive oxygen species; SOD: Superoxide dismutase.

## Competing interests

The authors declare they have no competing interests.

## Authors’ contributions

ETN and MO worked in the conception, methods, analysing results and revising it critically. CMA worked on the methods, data analysis and revising it critically. All three authors read and approved the final manuscript.

## Pre-publication history

The pre-publication history for this paper can be accessed here:

http://www.biomedcentral.com/1472-6882/12/247/prepub
